# Identification of CHD1L as an Important Regulator for Spermatogonial Stem Cell Survival and Self-Renewal

**DOI:** 10.1155/2016/4069543

**Published:** 2016-11-27

**Authors:** Shan-Shan Liu, Yin-Shan Bai, Li Feng, Wen-Wei Dong, Yang Li, Li-Ping Xu, Ning-Fang Ma

**Affiliations:** Department of Histology and Embryology, Guangzhou Medical University, Guangzhou, China

## Abstract

Chromodomain helicase/ATPase DNA binding protein 1-like gene (*Chd1l*) participates in chromatin-dependent processes, including transcriptional activation and DNA repair. In this study, we have found for the first time that Chd1l is mainly expressed in the testicular tissues of prepubertal and adult mice and colocalized with PLZF, OCT4, and GFR*α*1 in the neonatal mouse testis and THY1^+^ undifferentiated spermatogonia or spermatogonial stem cells (SSCs). Knockdown of endogenous* Chd1l* in cultured mouse undifferentiated SSCs inhibited the expression levels of* Oct4*,* Plzf*,* Gfrα1*, and* Pcna* genes, suppressed SSC colony formation, and reduced BrdU incorporation, while increasing SSC apoptosis. Moreover, the* Chd1l* gene expression is activated by GDNF in the cultured mouse SSCs, and the GDNF signaling pathway was modulated by endogenous levels of Chd1l; as demonstrated by the gene expression levels of GDNF, inducible transcripts* Etv5*,* Bcl6b*,* Pou3f*, and* Lhx1*, but not that of GDNF-independent gene,* Taf4b*, were significantly downregulated by Chd1l knockdown in mouse SSCs. Taken together, this study provides the first evidence to support the notion that* Chd1l* is an intrinsic and novel regulator for SSC survival and self-renewal, and it exerts such regulation at least partially through a GDNF signaling pathway.

## 1. Introduction

Chromodomain helicase/ATPase DNA binding protein 1-like gene (*Chd1l*), which encodes an unexplored chromatin remodeling factor with a similar domain to that of SNF2 gene family, is originally reported in liver cancer and functions as an oncogene [1]. Chromatin remodeling factors control most chromatin processes such as DNA replication and repair, gene transcription, homologous recombination, and cell cycling [2–7]. Evidence also suggests that some chromatin remodeling enzymes are involved in programming during development and that a number of ATP-dependent remodelers are necessary for maintaining vital function of embryonic stem cells (ESCs), such as self-renewal and differentiation [8]. Several genes that encode chromatin remodeling factors, including* Chd1l*, are found to be present in the inner cell mass of mouse blastosphere [9]. Moreover,* Chd1l* has been reported to associate with cell division [10] and regulate stem cell pluripotency through interplaying with PARP1 (poly(ADP-ribosyl)ation polymerase)/PARylation (poly(ADP-ribosyl)ation), during early developmental stage [11], implying a specific role for Chd1l in regulating stem cell pluripotency and/or embryonic development.

In an exploratory test on the expression of* Chd1l* in a panel of mouse tissues, we have observed that* Chd1l* mRNA is almost undetectable in numerous tissues but is highly expressed in testis tissues (unpublished data), which prompted us to speculate that* Chd1l* may be associated with spermatogenesis. The continual process of spermatogenesis depends on the self-renewal and differentiation potency of spermatogonial stem cells (SSCs) throughout the mammalian whole life. It is well known that the extrinsic cytokine, glial cell line-derived neurotrophic factor (GDNF), is required for SSCs proliferation and survival [12], and studies have reported that GDNF regulates the proliferation of SSCs via its receptor* GFRα1* [13]. Several other transcription factors (e.g.,* Nanog, Plzf, Bcl6b, Pou3f*, and* Etv5*) have also been reported to regulate SSC self-renewal [14–17]. In addition, some GDNF-independent factors (e.g.,* Plzf*,* Taf4b, Foxo1*, and* Pou5f1*) [18, 19] and the autocrine Hedgehog (Hh) signaling loop [20] have been recently suggested to sustain undifferentiated spermatogonia cells, suggesting that spermatogenesis is closely regulated by a fine and complicated regulatory network. To gain some insights into the functions of CHD1L in spermatogenesis, in this study, we have observed that CHD1L is coexpressed with the established markers of undifferentiated spermatogonia (GFR*α*1, PLZF) and the pluripotency factor OCT4 in newborn and adult testes of mice. Using a* Chd1l*-short hairpin RNA (shRNA) approach, we have further unraveled a novel functional role of Chd1l in SSC self-renewal and survival.

## 2. Materials and Methods

### 2.1. Animals

OCT4-GFP Tg C57BL/6J mice [21] were purchased from the Nanjing Biomedical Research Institute of Nanjing University. All procedures were performed according to institutional standard guidelines of the Committee on the Use of Live Animals in Teaching and Research of Guangzhou Medical University, Guangdong, China.

### 2.2. Immunohistochemistry of Mouse Testis Cross Sections

Immunohistochemical analysis was used to explore the expression of CHD1L in mouse testes. Testes were dissected from the mice, fixed in 4% paraformaldehyde for 12 h at 4°C, embedded in paraffin, and sectioned. Sodium citrate buffer (10 mM sodium citrate [pH 6.0]) was used for antigen retrieval by boiling the sections for 15 min. Endogenous peroxidase activity was blocked using 3% hydrogen peroxide for 10 min at room temperature. Sections were then blocked with 10% normal goat serum, followed by incubation with CHD1L antibody (Abcam, MA, USA) overnight at 4°C. After washing with PBS, the sections were incubated with horseradish peroxidase- (HRP-) conjugated goat anti-rabbit immunoglobulin G for 1 h at room temperature. The horseradish peroxidase detection kit (Zymed Laboratories, CA, USA) was used for color development followed by Mayer's hematoxylin counterstaining. Sections were examined under light microscopy. The antibodies and their concentrations are listed in Supplemental Table 2 in Supplementary Material available online at http://dx.doi.org/10.1155/2016/4069543.

### 2.3. Spermatogonial Stem Cell (SSC) Cultures

The mouse SSC line [22], a kind gift from Professor Fan LQ (Institute of Reproductive & Stem Cell Engineering, Central South University, Changsha, Hunan, China), was cultured in StemPro-34 SFM (Invitrogen, CA, USA), supplemented with 100 *μ*g/mL transferrin, 25 *μ*g/mL insulin, 30 nM sodium selenite, 2 mM L-glutamine, 20 ng/mL mouse epidermal growth factor (PeproTech Ltd., NJ, USA), 20 ng/mL mouse basic fibroblast growth factor (PeproTech Ltd.), and 15 ng/mL recombinant human GDNF (PeproTech Ltd.), on mouse embryonic fibroblasts (MEF) feeders, as described in a previous study [23]. All cultures were maintained at 34°C in a humidified 5% CO_2_ incubator. The medium was changed every 2-3 days and the cells were passaged at 7-day intervals with a ratio of 1 : 2-1 : 3.

### 2.4. Preparation of THY1^+^ Undifferentiated Spermatogonia

The segments of seminiferous tubules dissected from the testis of OCT4-GFP Tg C57BL/6J mouse at 5–7 days postpartum (dpp) were digested with Trypsin-EDTA (Invitrogen) and Accutase (Sigma, MO, USA). Single cell suspensions were layered on top of a 30% Percoll solution (Pharmacia, NJ, USA) and testis cells were enriched by centrifugation (300 ×g, 5 min). Undifferentiated spermatogonia were isolated from enriched testis cells by magnetic activated cell sorting (MACS) using CD90.2 (the thymus cell antigen 1, Thy1.2) antibody-conjugated microbeads (Miltenyi Biotec, Bergisch Gladbach, Germany). The THY1^+^ cells were subjected to immunostaining immediately or seeded at a density of 0.5 × 10^5^ cells per well (12-well culture plates) on mitomycin C-treated (Sigma) MEF feeders for further analysis.

### 2.5. Immunofluorescent Staining of Testis Cross Sections and Selected Germ Cells

The expression of CHD1L and other proteins in testis cross sections of OCT4-GFP Tg C57BL/6J mice or selected cells was determined using a standard procedure. Briefly, the testis sections or cells were incubated with respective primary antibodies diluted in PBS containing 0.5% BSA at 4°C overnight, followed by incubation with the appropriate Alexa Fluor dye-conjugated secondary antibodies (Invitrogen). The selected cells or testis sections were washed and incubated with DAPI (4,6-diamidino-2-phenylindole dihydrochloride hydrate) (Vector Laboratories, CA, USA) in PBS to label the cell nuclei, followed by examination using confocal laser scanning microscopy (Leica, Wetzlar, Germany). Normal goat or rabbit IgG were used as negative controls. The antibodies and their concentrations are listed in Supplemental Table 2. In some cases, image contrast was adjusted using Photoshop CS2 to better reflect our visual observations.

### 2.6. RNA Extraction and Analysis

Total testicular tissue RNA was extracted using RNeasy Mini Kit (Qiagen, CA, USA) according to the manufacturer's instructions. Reverse transcription was performed using a PrimeScript™ II 1st Strand cDNA Synthesis Kit (Takara Bio, Shiga, Japan). Quantitative real-time reverse transcription PCR (qRT-PCR) was performed with SYBR® Premix Ex Taq™ II (Takara Bio) in an ABI7500 thermocycler (Applied Biosystems, CA, USA). Relative quantification of gene expression was performed using the comparative Ct method (2^−ΔΔCt^). The results were expressed as fold induction over values obtained from the self-control GAPDH. All reactions were performed in triplicate. The primers are listed in Supplemental Table 1.

### 2.7. Western Blotting

Protein concentrations of mouse testes lysates were measured using the micro-BCA method (Pierce, Milwaukee, WI). The extracted proteins (25 *μ*g) were loaded on 12% SDS-polyacrylamide gels and then transferred onto polyvinylidene difluoride (PVDF) membranes (Hybond-P, Helsinki, Finland). The nonspecific binding was blocked with PBS buffer containing 0.1% Tween-20, 2% BSA, and 5% nonfat dry milk, and the blots were then incubated with anti-rabbit CHD1L (Abcam), anti-goat GFRΑ1 (Santa Cruz, Dallas, Texas), anti-goat PLZF (Santa Cruz), or anti-rabbit *β*-actin (Santa Cruz), respectively. After extensive washing with 0.1% Tween-20 in PBS, the blots were incubated with horseradish peroxidase-conjugated anti-goat (Santa Cruz) or anti-rabbit (Santa Cruz) IgG at room temperature for 1 h. Visualization was developed using ECL Plus Western Blotting Detection Kit (Amersham, Helsinki, Finland). Quantification of blot intensities was performed using Image Lab software (Bio-Rad, Hercules, California), according to the developer's protocol. *β*-Actin was immunoblotted and visualized as a loading control. The antibodies used and their concentrations are listed in Supplemental Table 2.

### 2.8. Chd1l Knockdown by Short Hairpin RNA in Mouse SSCs

The shRNAs targeting* Chd1l* mRNA were designed online (http://www.thermofisher.com/rnai.html): Chd1l shRNA#1, 5′-CAACTTACATATACTACTT-3′; Chd1l shRNA #2, 5′-GTTCATCTTCCACGAATTG-3′; a scrambled sequence that shared no homology with the mammalian genome was used as control. Successful cloning of these sequences into the hU6-MCS-CMV-Puromycin lentivector was confirmed by Sanger sequencing. The pGC-LV, pHelper 1.0, and pHelper 2.0 packaging system and the 293T cell line were used for the production of pseudoviral particles. The lentiviruses were produced in 293T cells by transient transfection of shRNA plasmids and above packaging plasmids into 293T cells according to standard protocols. Mouse SSCs were infected with CHD1L specific lentiviral particles at a ratio of 20 particles to 1 cell for 12 h in the presence of polybrene to improve transduction efficiency, and the infection efficiency was evaluated by GFP expression. The infected cells were maintained in mouse SSC culture medium. qRT-PCR analysis was performed to evaluate the effect of* Chd1l* downregulation.

### 2.9. Apoptosis and Flow Cytometry Analyses

Cells infected with scrambled shRNA or* Chd1l* shRNA expressing lentivirus were cultured 48 h later and subjected to standard cell apoptosis analysis. Briefly, cells were fixed and labeled with Annexin-V-fluorescein isothiocyanate and propidium iodide (PI) using the Annexin-V-FLUOS Staining Kit (BD Biosciences, CA, USA) according to the manufacturer's instructions, followed by flow cytometry analyses. The cells labeled with Annexin-V but without propidium iodide labeling were counted as apoptotic cells.

### 2.10. Colony Formation Assay

Mouse SSCs (1 × 10^5^/well) infected with scrambled control or Chd1l shRNA-expression lentivirus were reseeded into 6-well plates containing MEF feeder layer and incubated at 34°C in a humidified 5% CO_2_ incubator for 72 h. A cell cluster with a diameter greater than or equal to 50 *μ*m was defined as a colony. The numbers of colonies were evaluated using standard criteria. All data are the mean ± SD for three independent experiments. ^*∗*^ *P* < 0.05 compared with the control.

### 2.11. BrdU Incorporation Assays

Mouse SSCs were incubated with 40 *μ*M 5-bromo-2′-deoxyuridine (BrdU) (Sigma) for 30 min, and BrdU Cell Proliferation Assay Kit (Calbiochem, Darmstadt, Germany) was used to detect the BrdU incorporation according to the manufacturer's instructions. Nuclei were counterstained with DAPI. BrdU-positive and total nuclei were counted from 10 randomly selected fields under a 200x objective, and the percentage of BrdU-positive nuclei was calculated. All experiments were performed in triplicate and repeated three times.

### 2.12. Proliferation Assays

Mouse SSCs infected with scrambled control or Chd1l shRNA-expression lentiviruses were seeded at a density of 2000 cells per well in 96-well microtiter plates. After 24, 48, 72, and 96 h of culture, proliferation assays were performed using the CellTiter 96® Aqueous One Solution Assay (Promega, Madison, WI), according to the manufacturer's instructions.

### 2.13. Statistical Analyses

Results are presented as mean ± standard deviation (SD). Statistical analysis was performed using SPSS statistical software (SPSS, Inc., NY, USA). Two-tailed unpaired Student's *t*-test was used for comparisons between 2 groups, or one-way analysis of variance with post hoc Tukey test was applied when more than two groups were compared if the data display a normal distribution. *P* < 0.05 was considered statistically significant.

## 3. Results

### 3.1. Expression Patterns and Localization of Chd1l in Prepubertal and Adult Mouse Testes

**Figure 1:**
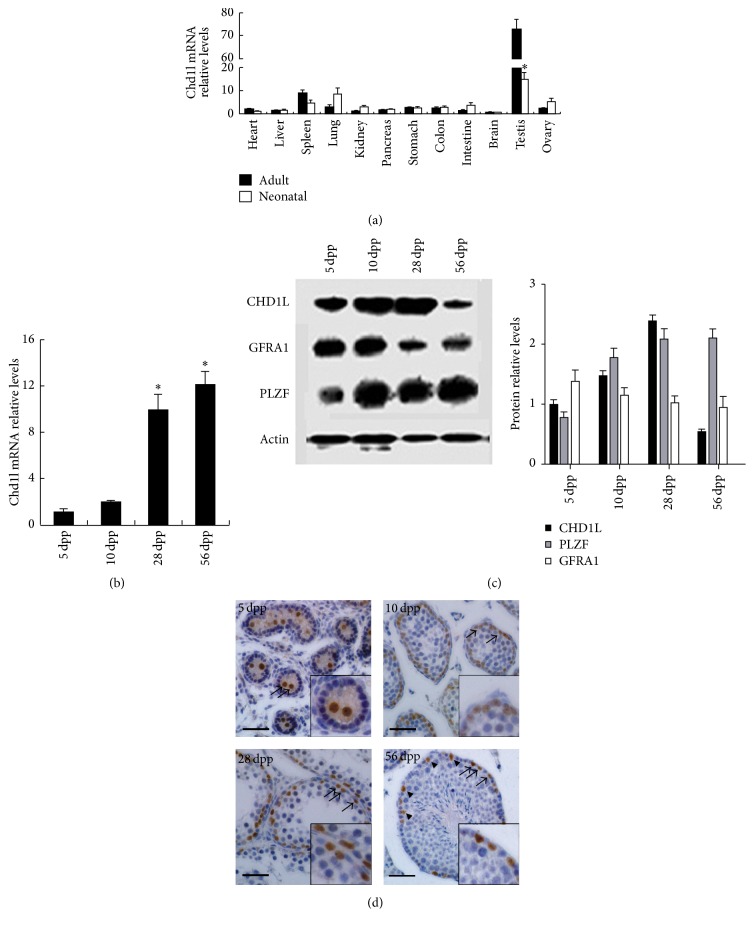
Expression of* Chd1l* in mouse testes. (a)* Chd1l* mRNA profiling in developing and adult mouse tissues, as assessed by quantitative RT-PCR analysis. Data presented here are mean ± SD of three animals (*n* = 3). ^*∗*^ *P* < 0.05 (versus adult tissues). (b) Expression of* Chd1l* in developing mouse testis. Data presented here are mean ± SD of three developing mouse testes (*n* = 3). ^*∗*^ *P* < 0.05 (versus 5-day testes). (c) Protein expression levels of CHD1L, GFRΑ1, and PLZF in developing mouse testis. Total proteins were harvested and subjected to Western Blot analyses. Representative images (left) and quantitative data (right) from three independent experiments (*n* = 3) were presented here. (d) Immunohistochemistry assays show that CHD1L is mainly expressed in the spermatogonia of the developing mouse testis at 5 dpp, 10 dpp, 28 dpp, and 56 dpp. Long arrows indicate the positive (brown) staining in testis sections, and the arrowheads indicate the positive nucleus of Sertoli cells in 56 dpp testis sections. Scale bars represent 100 *μ*m. Insets are magnified views. Representative images from three animals (*n* = 3) were presented here. Quantitative RT-PCR: quantitative reverse transcription-polymerase chain reaction; CHD1L: chromodomain helicase/ATPase DNA binding protein 1-like gene; PLZF: promyelocytic leukemia zinc finger; GFRΑ1: GDNF-family receptor *α*-1; SD: standard deviation; dpp: days postpartum.

To examine the expression profiles of* Chd1l* in various tissues, total RNAs were harvested from various mouse tissues and subjected to qRT-PCR analyses. We observed that* Chd1l* was highly expressed in neonatal and adult testes (Figure 1(a)). Data from qRT-PCR analyses showed that the expression levels of* Chd1l* transcript in postnatal mice testis were gradually increased from 5 dpp to adulthood (56 dpp). Specifically,* Chd1l* transcript is detectable at 5 dpp, significantly increased at 28 dpp, and maintained at a similar level thereafter (Figure 1(b)). CHD1L protein expression displayed a similar trend to its mRNA transcript before puberty as demonstrated in Western Blotting analyses, although a much lower level was observed at the adult stage (Figure 1(c)). The above data imply a role for CHD1L in mouse testis development and/or spermatogenesis.

To further explore the cellular localization of CHD1L in mouse testis, immunohistochemistry was used to examine CHD1L expression in paraffin embedding sections of different developmental stages mouse testes. We observed that CHD1L is expressed in all the prepubertal and adult mouse testes, and CHD1L-positive cells were randomly dispersed among the spermatogonia over the seminiferous tubule basal lamina, the stem cell zone of the testis [24]. No CHD1L-positive signal was detected in meiotic and postmeiotic germ cells (Figure 1(d)). Surprisingly, CHD1L staining was also found in the nuclei of Sertoli cells, which were clearly identifiable based on their defining characteristic of prominent nucleoli staining. No positive staining signal can be detected in the sections incubated with normal IgG controls. Thus, the immunostaining data provides another evidence to support the notion that* Chd1l* may be involved in testis development and/or spermatogenesis.

### 3.2. CHD1L Marks the Undifferentiated Spermatogonia Population

**Figure 2:**
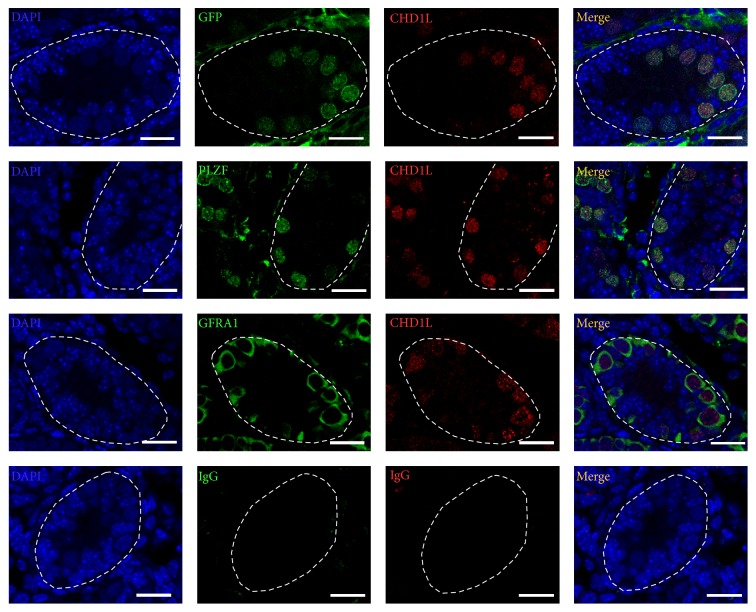
CHD1L is coexpressed with other SSC markers in undifferentiated spermatogonia cells. Immunofluorescence staining of mouse testicular tissues harvested at 5 dpp, sectioned, and subjected to immunofluorescence staining with antibodies against CHD1L (red), PLZF (green), GFRΑ1 (green), or GFP (green), respectively. The nuclei were visualized with DAPI (blue). The right panels are the merged images showing coexpression of CHD1L with PLZF, GFRΑ1, or GFP in most undifferentiated spermatogonia at 5 dpp. Normal goat or rabbit IgG were applied as negative controls (bottom row). Representative images from three animals (*n* = 3) were presented here. Scale bars represent 50 *μ*m. SSC: spermatogonial stem cell; dpp: days postpartum; PLZF: promyelocytic leukemia zinc finger; GFRΑ1: GDNF-family receptor *α*-1; DAPI: 4′,6-diamidino-2-phenylindole.

**Figure 3:**
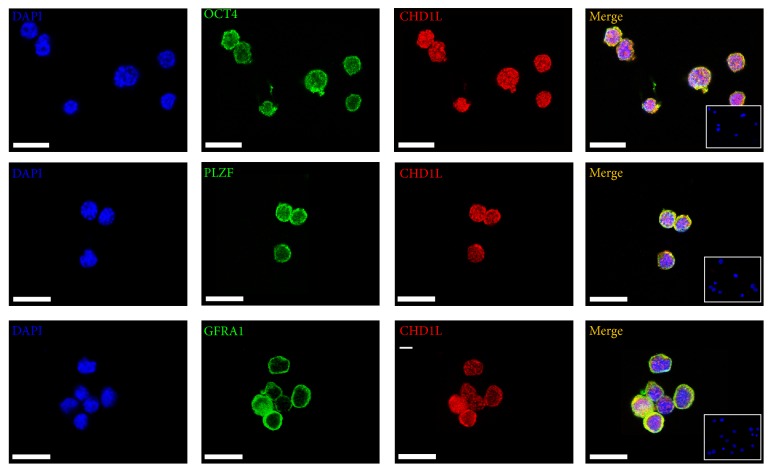
CHD1L is expressed in THY1^+^ SSCs. Immunofluorescence staining assays reveal the colocalization of CHD1L (red) with OCT4 (green), PLZF (green), and GFRΑ1 (green) in THY1^+^ SSCs. The THY1^+^ SSCs were isolated from a total spermatogonia pool, obtained from 5 dpp mouse testis, using THY1 conjugated immunomagnetic beads. Representative images from three independent experiments (*n* = 3) were presented here. The insert in the merged images is the IgG negative control. Scale bars represent 50 *μ*m. SSC: spermatogonial stem cell; dpp: days postpartum; THY1 (Thy-1): thymus cell antigen 1; GFRΑ1: GDNF-family receptor *α*-1.

The undifferentiated spermatogonia are the prevalent germ cell population before 5 dpp in the testes. To find out whether CHD1L-expressing germline cells are undifferentiated spermatogonia, immunofluorescent double-labeling was performed on the cross sections of neonatal mouse testis at 5 dpp. As expected, CHD1L expression overlapped substantially with the markers of undifferentiated spermatogonia (GFR*α*1 and PLZF) and the indicator of pluripotency factor OCT4 (GFP) in the spermatogonia of neonatal testis (Figure 2). It has been proven that THY1 (CD90.2) is a conserved marker of undifferentiated spermatogonia and selection of THY1-expressing testis cells results in enrichment of SSCs [25]. To further characterize the potential involvement of CHD1L in spermatogonia* in vitro*, THY1-positive cells were isolated from 5 dpp testis using MACS with conjugated beads and were analyzed using flow cytometry. We found that majority of enriched THY1-positive cells were positive for GFR *α*1 (Supplemental Figure 1). Importantly, data from double immunofluorescence staining assays revealed that almost all the enriched THY1-positive cells were positive for CHD1L, and CHD1L were coexpressed with other SSC markers (e.g., PLZF and GFR*α*1) and the pluripotent marker OCT4 in the enriched THY1^+^ cells (Figure 3). No positive signals were observed in the sections (Figure 2, bottom row) or cells (inserted figures in Figure 3) with IgG control, confirming the specific staining of the proteins. Taken together, the above data provide clear evidence to support the notion that CHD1L is expressed in undifferentiated spermatogonia of neonatal mice testes and/or SSCs.

### 3.3. CHD1L Plays an Important Role in Mouse SSC Proliferation

**Figure 4:**
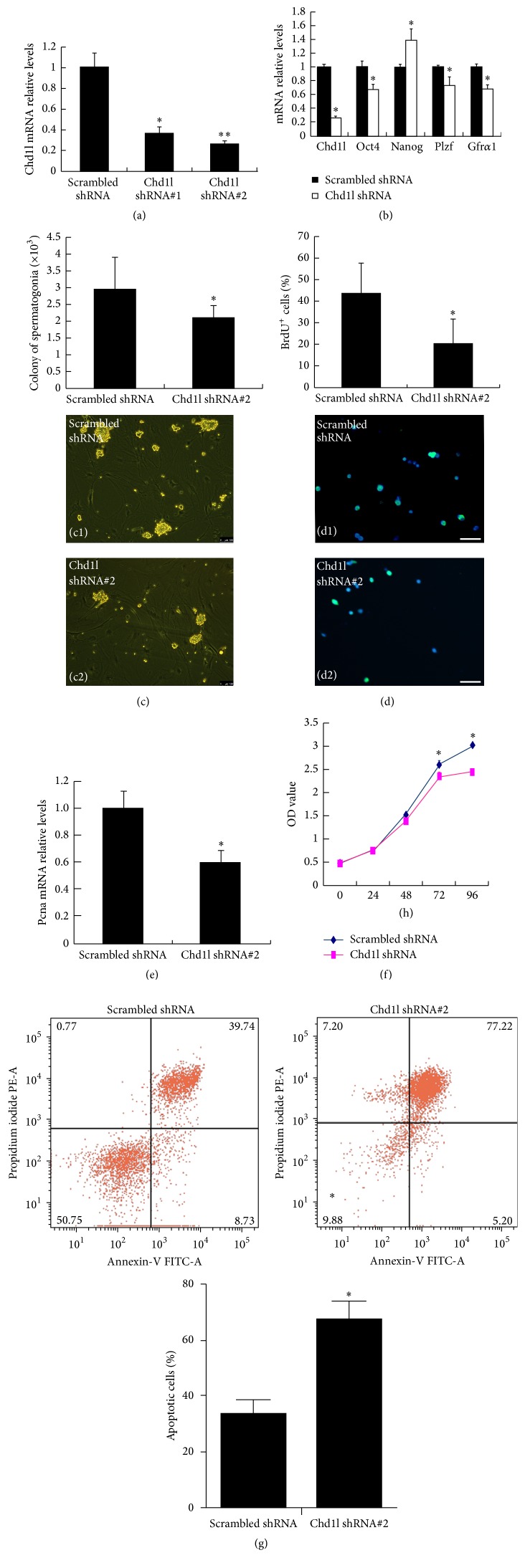
Chd1l modulates SSC gene expression, clonogenicity, proliferation, and apoptosis. (a)* Chd1l* gene knockdown in mouse SSCs. Mouse SSCs were infected with scrambled shRNA,* Chd11* shRNA #1, or* Chd11* shRNA #2 lentivirus. After 48 hours, total RNAs were harvested and subjected to quantitative RT-PCR analysis. (b)* Oct4*,* Plzf, Gfrα1*, and* Nanog* gene expressions are modulated by Chd1l knockdown in mouse SSCs. Mouse SSCs were infected with scrambled shRNA or Chd11 shRNA#2 lentivirus. After 48 hours, total RNAs were harvested and subjected to quantitative RT-PCR analysis with indicated primers. Data in (a) and (b) are the mean ± SD of three independent experiments. ^*∗*^ *P* < 0.05 compared with the control. (c) Clonogenicity of mouse SSCs was significantly inhibited by knockdown of* Chd1l*. Cell clusters whose diameters were greater than or equal to 50 *μ*m were defined as colonies. Bars = 200 *μ*m. Representative images (bottom) and quantitative data (up, colonies per well) from three independent experiments (*n* = 3) were presented here. ^*∗*^ *P* < 0.05 (versus the scrambled control). (d–f) Chd1l knockdown reduced mouse SSC proliferation. Mouse SSCs were infected with scrambled shRNA or Chd11 shRNA#2 lentivirus. Infected SSCs were cultured for further 48 hours (d and e), or the indicated times (f), followed by BrdU cell proliferation assays (d), quantitative RT-PCR analyses (e), or MTS assays (f), respectively. In panel (d), the representative images (bottom) and quantitative data (up, percentage of BrdU^+^ cells, green) from three independent experiments (*n* = 3) were presented. Bars = 50 *μ*m; ^*∗*^ *P* < 0.05 (versus scrambled control shRNA). Data presented in panels (e) and (f) are mean ± SD of three independent experiments. (g) Chd1l knockdown increased mouse SSC apoptosis. Apoptosis was compared between the scrambled shRNA control and* Chd1l* shRNA#2 cells using flow cytometry. The cells labeled with Annexin-V but without propidium iodide labeling were counted as apoptotic cells. *x*-axis: intensity of Annexin-V, relative units; *y*-axis: intensity of propidium iodide incorporation, relative units. The percentage of apoptotic cells is the mean ± SD of three independent experiments. ^*∗*^ *P* < 0.05 compared with the control. shRNA: short hairpin RNA; BrdU: 5-bromo-2′-deoxyuridine; SSCs: spermatogonial stem cells; SD: standard deviation. ^*∗∗*^ *P* < 0.01 compared with the control.

An* in vitro* culture system for undifferentiated spermatogonia has been recently established using a SSC cell line [22]. To investigate whether CHD1L is essential for SSC survival and other functions, Chd1l specific shRNA lentiviruses were generated and used for Chd1l knockdown in mouse SSCs. qRT-PCR analyses confirmed that the endogenous expression level of Chd1l was significantly downregulated by both Chd1l specific shRNA lentiviruses with #2 shRNA providing a better knockdown efficiency (Figure 4(a)). Accordingly, #2 Chd1l shRNA lentivirus was used in the following knockdown experiments. Interestingly, knockdown of Chd1l in mouse SSCs resulted in a decreased level of* Oct4*,* Plzf*, and* Gfrα1*, but an increased level of* Nanog* (Figure 4(b)), suggesting that Chd1l specifically regulates these genes in mouse SSCs. It has been well known that clonogenicity is an important characteristic of stem cells. To further explore a potential role of Chd1l in mouse SSC function, colony formation assay was conducted in the SSCs infected with scrambled control and Chd1l shRNA lentivirus. Data shown in Figure 4(c) showed that the ability to form colony of the SSCs infected with Chd1l shRNA lentivirus was much lower than that of cells treated with control scrambled shRNA lentivirus. Moreover, BrdU incorporation assays revealed that Chd1l knockdown significantly decreased SSCs proliferation (Figure 4(d)), which was further confirmed by a lower expression level of Pcna mRNA and lower cell survivability in the SSCs infected with Chd1l lentivirus as demonstrated in qRT-PCR analyses (Figure 4(e)) and MTS assays (Figure 4(f)), respectively. Finally, cell apoptosis was conducted to determine whether Chd1l plays a role in SSCs apoptosis.  Figure 4(g) showed that inhibition of endogenous Chd1l expression level in SSCs dramatically increased SSC apoptosis (33.25 ± 5.69% versus 67.34 ± 6.81% of apoptotic cells). These data provide compelling evidence to suggest that* Chd1l* plays an important role in regulation of SSC gene expression, clonogenicity, proliferation, and apoptosis.

### 3.4. Chd1l Is Regulated by and Involved in GDNF Signaling Pathway in Mouse SSCs

**Figure 5:**
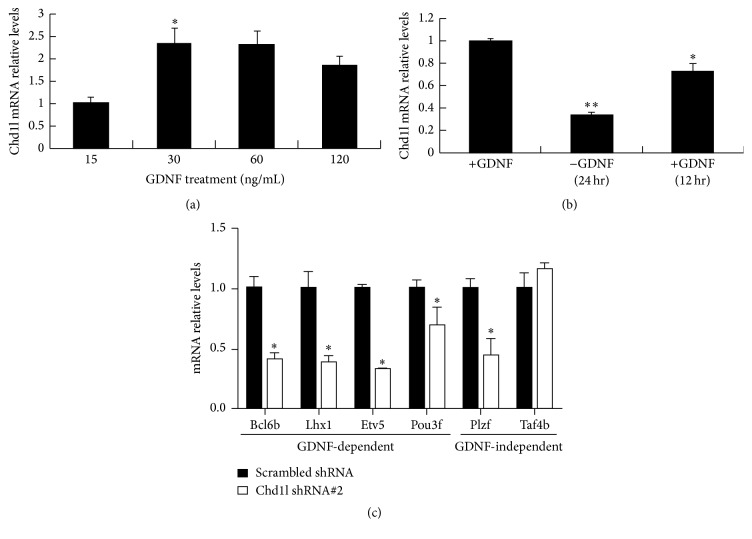
Chd1l is involved in GDNF signaling pathway. (a) Effects of supplementation with higher levels of GDNF on* Chd1l* gene expression in mouse SSCs. (b) Effects of GDNF withdrawal or replacement on* Chd1l* gene expression in mouse SSCs. Mouse SSCs were normally maintained in the culture medium supplemented with 15 ng/mL GDNF. GDNF was withdrawn from the culture medium for 24 hours, followed by GDNF restimulation for another 12 hours. Total RNAs were harvested at the indicated time and subjected to quantitative RT-PCR analyses. (c) GDNF-dependent downstream genes are regulated by Chd1l in mouse SSCs. Mouse SSCs were infected with scrambled shRNA or Chd11 shRNA#2 lentivirus. After 48 hours, total RNAs were harvested and subjected to quantitative RT-PCR analyses with the indicated primers. Data presented here are the mean ± SD of three independent experiments. ^*∗*^ *P* < 0.05 (versus normal SSC culture medium in panels (a) and (b); versus scrambled control shRNA in panel (c)), and ^*∗∗*^ *P* < 0.01 (GDNF restimulation versus GDNF withdrawal). SSCs: spermatogonial stem cells; GDNF: glial cell line-derived neurotrophic factor.

The response to extrinsic GDNF stimulation is a vital mechanism of SSC survival and self-renewal. Our above data suggests that Chd1l plays a functional role in SSC survival and self-renewal. We wondered whether Chd1l expression was modulated by GDNF. As expected, supplementing a higher amount of GDNF to mouse SSCs significantly upregulated Chd1l gene expression levels (Figure 5(a)), while GDNF withdrawal from culture medium resulted in a decreased Chd1l expression level, and such inhibitory effect was almost abolished upon GDNF restimulation (Figure 5(b)), indicating that Chd1l is closely regulated by GDNF in mouse SSCs. Given that GDNF promotes SSC self-renewal and stimulates antiapoptotic pathways that support SSC survival [26], we hypothesized that CHD1L mediated SSC survival and self-renewal might be through GDNF signaling pathway. Expectedly, we observed that GDNF-dependent transcripts (e.g.,* Etv5*,* Bcl6b*,* Pou3f*, and* Lhx1*), but not the known GDNF-independent transcript* (Taf4b)*, were significantly inhibited by Chd1l knockdown in SSCs (Figure 5(c)), supporting a regulatory role of Chd1l in GDNF signaling.

## 4. Discussion

CHD1L plays a vital role in keeping the chromosome integrity and promoting DNA repair [2]. Functional studies have proven that overexpression of CHD1L promotes G1/S phase transition, inhibits apoptosis, and promotes cell proliferation through inhibiting p53 signaling in solid tumors [1]. Further exploration of this tumor driver gene may lead to a better understanding of its molecular features and biological functions. A recent study has suggested a spatial and temporal expression pattern of* Chd1l* during ESC differentiation and a vital role for this gene in preimplantation and the earliest phase of cell division of mammalian development [10]. In the present study,* Chd1l* was found to be highly expressed in the testis, implying a testes-specific role for CHD1L. Interestingly, the trend of CHD1L protein expression did not coincide with its mRNA at the adult stage, suggesting that some unknown posttranscriptional mechanisms are involved in the regulation of CHD1L protein at adult stages. Immunostaining analyses of sections from testes of 5 dpp mice showed that the cells positive for CHD1L are mainly localized in undifferentiated spermatogonia, indicating a role for CHD1L in spermatogenesis.

Spermatogonial stem cells (SSCs) belong to adult stem cells which produce mature gametes in the testes throughout the male's life. Many transcription factors have been implicated in SSCs self-renewal. Undifferentiated spermatogonia, comprised of As, Apr, and Aal spermatogonia among type A spermatogonia, have all the stem cell characteristics including the self-renewal ability and differentiation potential [27]. PLZF and GFR*α*1 have been reported to be expressed in undifferentiated spermatogonia and function as specific regulators to promote the self-renewal of the stem cell pool in the testis [15, 28, 29]. To further confirm the nature of CHD1L-expressing cells during spermatogenesis, OCT4-GFP transgenic mice were used in this study. Double-labeling the cells in the undifferentiated spermatogonia of mouse testes with GFP (OCT4) and CHD1L revealed that almost all of the CHD1L-expression cells were positive for OCT4 (Figures 2 and 3), indicating that CHD1L is an important and useful marker for undifferentiated spermatogonia. Such a notion has been further supported by the evidence that CHD1L-expressing cells can also regulate the SSC specific transcription factors, PLZF and GFR*α*1, and ES cells specific transcription factors, Oct4 and Nanog (Figure 4). A parallel expression pattern between these specific transcription factors and Chd1l provided another convincing proof besides that of Nanog, which is increased while Oct4 expression is decreased upon knockdown of Chd1l. In mouse ES cells, Oct4, Sox2, and Nanog have been proven to be the important regulators for pluripotency [30–32]. The continued expression of Oct4 is confirmed to be essential for Nanog-mediated self-renewal; however, Oct4 is not requisite for Nanog expression. Similarly, evidence indicated that Oct4 expression is not increased in Nanog overexpression [33]. The increased expression of Oct4 causes ES cells to differentiate whereas increased Nanog prevents differentiation [34]. Hence, it may be inferred from these studies that Nanog and other SSC specific transcription factors probably act in concert rather than in series.

CHD1L has been reported to promote tumor cell proliferation through modulating p53 expression and interacting with various proteins (e.g., Nur77) [35]. A previous study reported a role for CHD1L in regulation of cell apoptosis through controlling the translocation of Nur77 protein from nucleus to mitochondria and the release of impaired cytochrome* c* [4]. Consistent with this finding, we found that knocking down Chd1l by shRNA in cultured mouse SSCs resulted in a lower level of BrdU incorporation and proliferation rate, a reduced expression level of* Pcna*, decreased clonogenicity, and increased cell apoptosis, indicating an essential role of CHD1L in spermatogonial proliferation and self-renewal. Interestingly, we have noted that CHD1L is also expressed in some Sertoli cells (Figure 1(d)). SSCs are the foundation of spermatogenesis and male fertility, which is initiated in fetal and early postnatal life, and the Sertoli cells play a significant role in continuous sperm production [36]. Dynamic changes in the proliferation of Sertoli cells appear around puberty and mature stable state, which is regulated by a complicated series of endocrine, paracrine, and autocrine factors [26, 37, 38]. Our finding that some of the Sertoli cells are positive for CHD1L may suggest a role for CHD1L in Sertoli cell functions, which remains to be elucidated in our future studies.

Previous studies have provided evidence to suggest that GDNF signaling is essential for SSC survival and fate determination [39]. In this aspect, our data show that CHD1L expression in SSCs is closely modulated by GDNF. Specifically, supplementing a higher concentration of GDNF to cultured SSCs induced an increase of CHD1L gene expression, while GDNF withdrawal from the culture medium resulted in inhibition of CHD1L expression. Importantly, the expression level of CHD1L returned a comparable level to that of cells under normal culture condition when resupplying GDNF to the GDNF-starved SSCs. Moreover, inhibiting the endogenous expression level of* Chd1l* in SSCs downregulated the expressions of* Etv5*,* Bcl6b*,* Pou3f*, and* Lhx1*, which are GDNF-dependent factors, while it plays no significant role in the regulation of the GDNF-independent factor* Taf4b*, suggesting that CHD1L is a major component of GDNF signaling pathway. It is worth noting that one of the GDNF-independent factors,* Plzf,* is downregulated by CHD1L knockdown. As a proven GDNF-independent factor, PLZF has been suggested to play an important role in maintaining the SSC pool. Our data raises the possibility that CHD1L regulates the GDNF-dependent and GDNF-independent downstream genes through a different molecular mechanism, which warrants further investigation. Taken together, in the current study, we have demonstrated for the first time that CHD1L is an important and useful marker for identifying and characterizing SSCs or spermatogonia* in vivo* and* in vitro*, and CHD1L plays a critical role in the regulation of SSC survival and self-renewal. Moreover, we also provided the first evidence to support the notion that CHD1L exerts such functions likely through modulating GDNF signaling in SSCs. Our data provide new insights into the spermatogenesis.

## 5. Conclusion

In this study, we showed that* Chd1l* is expressed in undifferentiated spermatogonial cells and is colocalized with Oct4, Plzf, and Gfr*α*1 in neonatal mice testis and m-SSCs. Expression of* Chd1l* is regulated by GDNF in cultured m-SSCs, and knocking down* Chd1l* by shRNA inhibited the expression of Oct4, Plzf, and Gfr*α*1 in m-SSCs and decreased the proliferation of the cultured m-SSCs. The expression of key components in the GDNF pathway also declined when* Chd1l* expression was inhibited. The results provided evidence that CHD1L is an intrinsic regulator of GDNF-induced survival and self-renewal of mouse SSCs.

## Supplementary Material

Supplementary Table 1: Primers used for quantitative RT-PCR. Supplementary Table 2: Antibodies used in experiment. Supplementary S1: Flow cytometric analysis of GFRα1 expression CD90+ SSCs. The undifferentiated spermatogonial stem cells were isolated from mouse testis at 5 dpp by using magnetic-activated cell sorting with CD90 conjugated microbeads. Isolated CD90+ cells were subjected to flow cytometry analyses using antibody against GFRα1 to examine GFRα1 expression on CD90+ SSCs. Left, CD90+ cells were gated and counted (green dot). Right, GFRα1 expression in CD90+ SSCs.

## Additional Points

*Summary. Chd1l* controls spermatogonial stem cell self-renewal and survival via GDNF pathway.
