# Supplementary material for: Identification of CHD1L as an Important Regulator for Spermatogonial Stem Cell Survival and Self-Renewal
Source: Stem Cells Int. 2016 Nov 27;2016:4069543. doi: 10.1155/2016/4069543 (PMC5149700; doi:10.1155/2016/4069543)
Supplement: Supplementary file 1 — Supplementary Table 1: Primers used for quantitative RT-PCR. Supplementary Table 2: Antibodies used in experiment. Supplementary S1: Flow cytometric analysis of GFRα1 expression CD90+ SSCs. The undifferentiated spermatogonial stem cells were isolated from mouse testis at 5 dpp by using magnetic-activated cell sorting with CD90 conjugated microbeads. Isolated CD90+ cells were subjected to flow cytometry analyses using antibody against GFRα1 to examine GFRα1 expression on CD90+ SSCs. Left, CD90+ cells were gated and counted (green dot). Right, GFRα1 expression in CD90+ SSCs. [file 4069543.f1.pdf]

Supplemental Table 1 Primers used for quantitative RT-PCR

primer	Sequence	Product size (bp)
GAPDH forward	5′-GCCTCAAGATCAGCAAT-3′	310
GAPDH reversed	5′-AGGTCCACCACTGACACGTT-3′	
Chd1l forward	5’-GACCTGAGTTTGGGTGATG-3’	260
Chd1 reversed	5’-CGGATAAGTCGTTCGGTA-3’	
Oct4 forward	5′-GGCGTTCTCTTTGGAAAGGTGTTC-3′	302
Oct4 reversed	5′-CTCGAACCACATCCTTCTCT-3′	
Nanog forward	5′-GCTGAGATGCCTCACACGGAG-3′	163
Nanog reversed	5′-TCTGTTTCTTGACTGGGACCTTGTC-3′	
Plzf forward	5′-GTGCCTCGCCATACCAGTGTAC-3′	262
Plzf reversed	5′-CCCCTTTTCTTTTCTGTTATTCTTTT-3′	
Gfrα1 forward	5′-GATTACGGAAAGGATGGTCTCG-3′	348
Gfrα1 reversed	5′-GTTTGGAATTAGCCCTGTAGCA-3′	
Bcl6b forward	5′-AAGCCGTATAAGTGTGAGACG-3′	257
Bcl6b reversed	5′-AGAATGTGGTAGTGCAC-3′	
Lhx1 forward	5′-TTCCCGCAGAACCTGAAGAT-3′	195
Lhx1 reversed	5′-CCCCAGAAATGCCAGATTAC-3′	
Etv5 forward	5′-AACTTGGTGCTTCATGCTCC-3′	281
Etv5 reversed	5′-ACTTAGCACCAAGAGCCTGC-3′	
Pou3f forward	5′-GGCACATTTATTCACCAAGACCAG-3′	159
Pou3f reversed	5′-TGTGGACCTCAGCAGCATTTGGAT-3′	

Taf4b forward	5′-CACTGCCCTTCCTAAACTCGG-3′	196
Taf4b reversed	5'-TTACTTGTGGTCTTGGCTCCTGT-3'	

Note: All primers were designed with Beacon designer 2.

Supplemental Table 2 Antibodies used in experiment

Antibody	Specificity	Dilution	Company
CHD1L	Rabbit	1:50 / 1:300	Abcam
PLZF	mouse	1:100 / 1:500	Santa Cruz Biotechnology
GFRA1	mouse	1:100 / 1:500	Santa Cruz Biotechnology
β-actin	Rabbit	- / 1:1000	Santa Cruz Biotechnology
Goat anti-mouse IgG	Goat	1:200 / 1:5000	Santa Cruz Biotechnology
goat anti-rabbit IgG	Goat	1:200 / 1:5000	Santa Cruz Biotechnology
Alexa Fluor 488-conjugated goat anti-rabbit IgG	Goat	1:200 / -	Jackson Immuno Research
Alexa Fluor 594-conjugated goat anti-mouse IgG	Goat	1:200 / -	Jackson Immuno Research

Dilution (Immunostaining/Western Blot)

Supplemental Figure 1

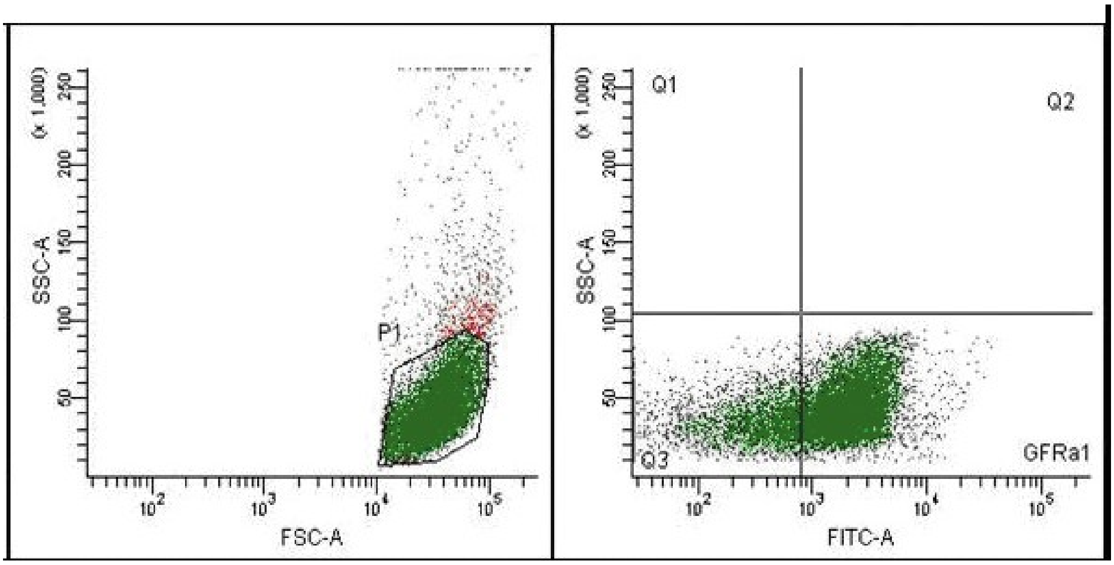

Supplemental Figure legend

Figure. S1 Flow cytometric analysis of GFRα1 expression CD90$^+$ SSCs**.** The undifferentiated spermatogonial stem cells were isolated from mouse testis at 5 dpp by using magnetic-activated cell sorting with CD90 conjugated microbeads. Isolated CD90$^+$ cells were subjected to flow cytometry analyses using antibody against GFRα1to examine GFRα1 expression on CD90$^+$ SSCs. Left, CD90$^+$ cells were gated and counted (green dot). Right, GFRα1 expression in CD90$^+$ SSCs.
